# Multidimensional Phononic Bandgaps in Three-Dimensional Lattices for Additive Manufacturing

**DOI:** 10.3390/ma12111878

**Published:** 2019-06-11

**Authors:** Waiel Elmadih, Wahyudin P. Syam, Ian Maskery, Dimitrios Chronopoulos, Richard Leach

**Affiliations:** 1 Manufacturing Metrology Team, Faculty of Engineering, University of Nottingham, Nottingham NG8 1BB, UK; Wahyudin.Syam@nottingham.ac.uk (W.P.S.); Richard.Leach@nottingham.ac.uk (R.L.); 2 Centre for Additive Manufacturing, Faculty of Engineering, University of Nottingham, Nottingham NG8 1BB, UK; Ian.Maskery@nottingham.ac.uk; 3 Institute for Aerospace Technology & Composites Research Group, Faculty of Engineering, University of Nottingham, Nottingham NG8 1BB, UK; Dimitrios.Chronopoulos@nottingham.ac.uk

**Keywords:** lattice structures, bandgaps, vibration isolation

## Abstract

We report on numerical modelling of three-dimensional lattice structures designed to provide phononic bandgaps. The examined lattice structures rely on two distinct mechanisms for bandgap formation: the destructive interference of elastic waves and internal resonance. Further to the effect of lattice type on the development of phononic bandgaps, we also present the effect of volume fraction, which enables the designer to control the frequency range over which the bandgaps exist. The bandgaps were identified from dispersion curves obtained using a finite element wave propagation modelling technique that provides high computational efficiency and high wave modelling accuracy. We show that lattice structures employing internal resonance can provide transmissibility reduction of longitudinal waves of up to −103 dB. Paired with the manufacturing freedom and material choice of additive manufacturing, the examined lattice structures can be tailored for use in wide-ranging applications including machine design, isolation and support platforms, metrology frames, aerospace and automobile applications, and biomedical devices.

## 1. Introduction

The design freedom of additive manufacturing (AM) enables the production of complex structures with tailorable properties for various applications. One of these properties is vibration isolation, which is conventionally achieved using high-mass structures to damp the response of different mechanical excitations. The high-mass approach ensures that the resonant frequency of the structure is higher than the operational frequency of the application of interest. However, this does not restrict the propagation of elastic waves and, therefore, limits the extent of the achievable vibration isolation. AM phononic bandgap (BG) structures based on repeating lattice unit cells provide a new approach to vibration isolation, with low vibration transmission and high tailorability, without the cost of high-mass structures.

**Figure 1:**
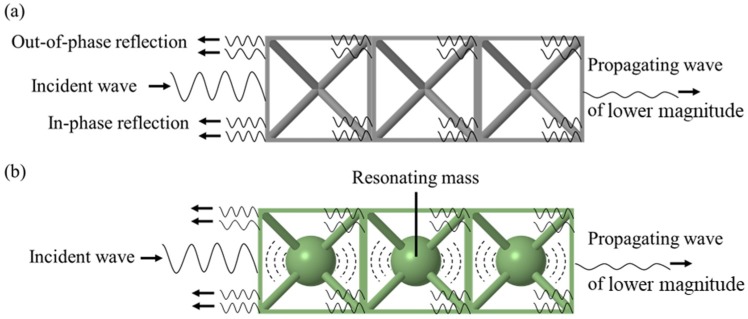
Illustration of the bandgap mechanism in (**a**) Bragg-scattering lattices and (**b**) internal resonance lattices.

Phononic BG structures are those in which elastic wave propagation is restricted at certain frequencies. These have received considerable attention recently, mainly for their ability to provide enhanced vibration isolation compared to that resulting from conventional design approaches. The concept of BGs emerged from solid-state physics, with recent use in electronic systems [1,2,3], photonics [4,5,6,7] and phononic structures [8,9,10,11,12,13]. BGs generally result from Bragg scattering, in which transmitted and reflected waves within a periodic medium undergo destructive interference [14,15,16,17,18,19]. The BG frequencies depend on the geometry and size of the repeating lattice unit cell [20]. BGs can also arise through a different phenomenon: internal resonance, where the energy of elastic waves of certain frequencies is absorbed by internal resonators embedded in the structure [21,22,23,24,25]. These BG formation mechanisms are illustrated in Figure 1. In Figure 1a, elastic waves are reflected due to the difference in mechanical impedance within the lattice structure. These waves destructively interfere with the propagating wave when they are out of phase with one another, leading to a Bragg-scattering BG. In Figure 1b, another BG formation mechanism co-exists alongside Bragg scattering; the energy of the elastic waves is absorbed by a resonating mass in each unit cell to create an internal resonance BG. For both BG formation mechanisms, increasing the lattice periodicity leads to higher attenuation of transmitted waves within the BG frequency range [19,20].

Internal resonance BGs in three-dimensional (3D) AM lattices were previously studied by Lucklum et al. [26] and D’Alessandro et al. [27]. Zhang et al. [28] studied beam structures with periodically embedded resonators. Liu et al. [29] investigated structures with solid cores and a silicone rubber coating. Wang et al. [25] presented holey phononic crystals with resonators, while Matlack et al. [15] presented an AM polymer lattice with implanted metallic resonators. Various AM Bragg-scattering BG lattices have also been studied; for example, Warmuth et al. [30] manufactured and tested BG lattices based on interconnected struts, Wormser et al. [31] experimentally identified BGs in lattices similar to those of Warmuth et al. [30], and Lucklum et al. [32] presented strut-based lattices at the millimetre scale. Non strut-based AM BG lattices can be seen in the work of Elmadih et al. [18] and Abueidda et al. [8]; in both these cases, BG structures were obtained using lattices based on triply periodic minimal surface (TPMS) equations. Non strut-based AM BG lattices can also be seen in the ceramic lattice work of Kruisová et al. [33] and Ampatzidis et al. [17].

Research on AM TPMS lattices has mainly focused on their mechanical and heat dissipation properties [34,35,36,37,38,39], though Elmadih et al. [18] have predicted the development of one-dimensional (1D) BGs in gyroid TPMS lattices. However, to the best of our knowledge, the ability of gyroid TPMS lattices to provide multidimensional BGs has not been studied, and this is critical if they are to be employed in general cases for vibration isolation. Because of their high specific stiffness and large surface-to-volume ratio, gyroid-based lattices could see use in the aerospace sector, where heat exchangers are commonly integrated into structural elements [40]. Further applications exist as support structures in the automotive and aerospace sectors, where vibration isolation and impact resistance are essential properties [41]. 

Previous work on the body-centered cubic (BCC) lattice showed that it has good manufacturability from polyamide material with laser powder bed fusion (L-PBF) [42] and from metals using selective laser melting [43]. The BCC lattice has high strength-to-weight ratio in comparison with other strut-based lattices, for example those comprising simple cubic (SC) and face-centered cubic (FCC) cells [44]. BCC lattices with additional reinforcement struts along a single direction were studied by Leary et al. [44], who concluded that these lattices have higher impact energy absorption than the conventional BCC design. To the best of our knowledge, the propensity for BCC lattices with reinforcement struts to form BGs has not been studied. Syam et al. [42] determined the natural frequencies of BCC lattices with additional reinforcement struts in the x, y and z directions (designated as BCC_xyz_) for vibration isolation purposes, but did not model the dispersion curves (DCs) of the lattice, and did not report on the effect of the lattice volume fraction on achieving vibration isolation. Lu et al. [13] and Hsieh et al. [45] independently predicted the DCs of multimaterial BCC lattice designs, but to date there have been no reports on the manufacturability or performance of these designs. In comparison to single-material AM, which is well-established, multimaterial AM currently requires manual assembly (such as in the lattice work of Matlack et al. [15]), requires support structures that constrain the design of the part, necessitates post-processing (such as in the work of Choi et al. [46]), and is limited to a small range of materials. 

The novelty of this work is in the examination of multidimensional BGs in three types of single material lattice, which have not been studied previously. These lattices are the BCC_xyz_, the network gyroid (gyroid TPMS) and a modified BCC_xyz_ lattice with internal resonators (res-BCC_xyz_). The existence of multidimensional phononic BGs would add vibration isolation to the existing panoply of controllable mechanical performance of the examined lattice structures [34,37,38,43,44]; thus enabling them to simultaneously fulfill various mechanical and vibrational functions. The BGs of the examined lattices were identified from their respective DCs and predicted using a multidimensional finite element (FE) wave propagation modelling method. 

The DC computational method was developed as an expansion of the 1D and two-dimensional (2D) FE techniques used elsewhere [14,15,16,17,18], and is described in Section 2.2. In Section 2.1, the designs and structural parameters of the examined lattices are presented. In Section 3.2, the multidimensional DCs of the lattices are presented. In Section 3.3, the results are discussed with respect to a selection of relevant findings from the literature, while ‘tuning’ of the lattice BGs through volume fraction control is discussed in Section 3.3. Lastly, the evolution of the predicted BGs with physically manufacturable periodicity, as opposed to the infinite periodicity of computational models, is presented in Section 3.4. 

## 2. Methods

### 2.1. Lattice Design

**Figure 2:**
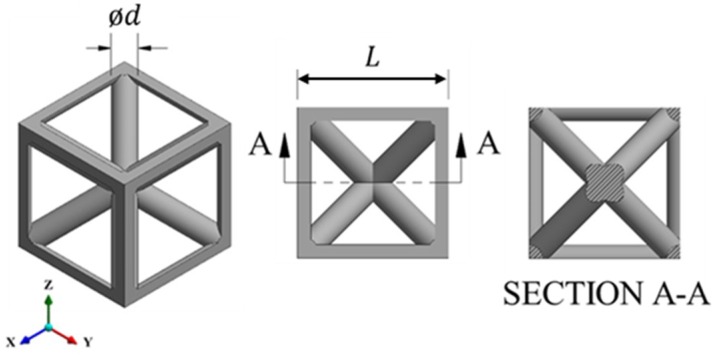
BCC_xyz_ lattice unit cell as designed in CAD with strut diameter *d* and cell size *L*.

The BCC_xyz_ unit cell, shown in Figure 2, was designed using the strut-based lattice design equations presented in our previous work [47]. In designing BCC_xyz_ lattice structures for this study, we considered a range of volume fractions from 5% to 30%. The corresponding ratios of strut diameter *d* to cell width *L* are provided in Table 1. A change in the d/L ratio leads to a change in the volume fraction of the lattice.

**Figure 3:**
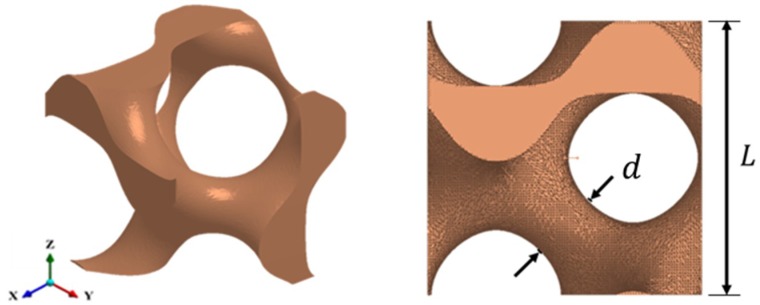
Gyroid TPMS unit cell with minimum thickness d and cell size L.

The unit cell of the gyroid TPMS lattice, shown in Figure 3, was produced using modelling software developed at the University of Nottingham [48]. The design equations for gyroid TPMS lattices can be found in the work of Maskery et al. [35]. The design information, expressed again as the ratio of strut thickness to cell width, for gyroid TPMS unit cells of different volume fractions, is available in Table 2. In this case, *d* represents the diameter of the unit cell’s solid region at its narrowest point.

**Figure 4:**
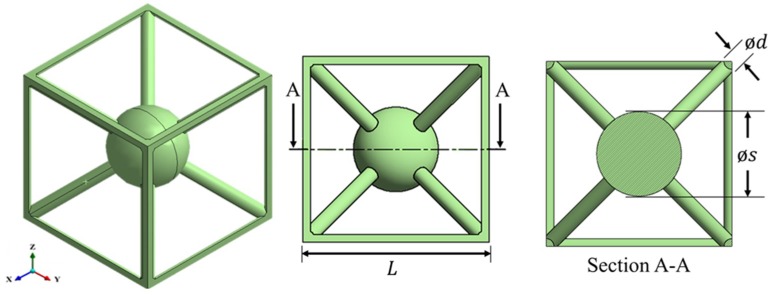
Res-BCC_xyz_ unit cell as designed in CAD with strut diameter d, spherical mass of diameter s and cell size L.

A solid spherical mass of diameter s was added to the centre of the BCC_xyz_ unit cell to create the res-BCC_xyz_ unit cell, as shown in Figure 4. The outer scaffold of the res-BCC_xyz_ is a 5% volume fraction BCC_xyz_ cell. Although different scaffolds can be considered using the same concept, the 5% volume fraction BCC_xyz_ lattice features a central void of sufficient size to host spherical masses with a wide range of sizes. The design information for the res-BCC_xyz_ unit cells at different volume fractions is presented in Table 3.

### 2.2. Bandgap Prediction 

**Figure 5:**
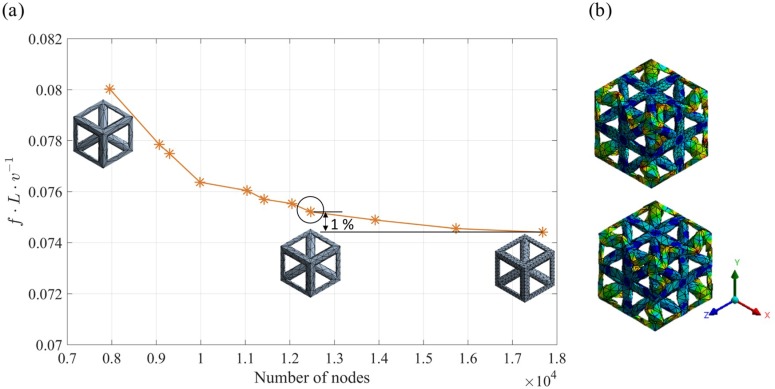
(**a**) Convergence results of the first natural frequency with respect to the mesh density of a 2 × 2 × 2 BCC_xyz_ lattice (converged mesh density is highlighted) and (**b**) comparison of high frequency vibration modes (existing above a normalised frequency of 0.3) of converged mesh (bottom) and finer mesh (top).

A FE-based wave propagation method was used to calculate the elastic dispersion curves of the lattice structures described in Section 2.1. In comparison to other dispersion calculation methods, for example, plain wave expansion (PWE) [10], finite difference time domain (FDTD) [49], wavelet [50,51], and multiple scattering theory (MST) [52], the FE method provides higher compuational efficiency and greater wave modelling accuracy [53,54]. The FE method incorporates Bloch’s theorem, which governs the displacement of the FE nodes, and Floquet boundary conditions, which approximate the infinite tessellation of the unit cell in 3D [55]. The calculations used 3D lattice models, with three degrees of freedom (DOFs) at each node, to capture all the possible modes of vibration. BCC_xyz_ and res-BCC_xyz_ unit cells were meshed in ANSYS simulation software using tetrahedral elements, and gyroid TPMS unit cells were meshed using hexagonal elements. Mesh convergence was determined through examination of the structure’s first natural frequency, which in each case was found to be well converged with respect to the mesh density (see Figure 5a). To ensure convergence of high frequency results (particularly above a normalised frequency of 0.3), a high frequency vibration mode of the converged mesh was compared to that of a finer mesh. The results, shown in Figure 5b, showed minimal discrepancies in the vibration mode and frequency.

**Figure 6:**
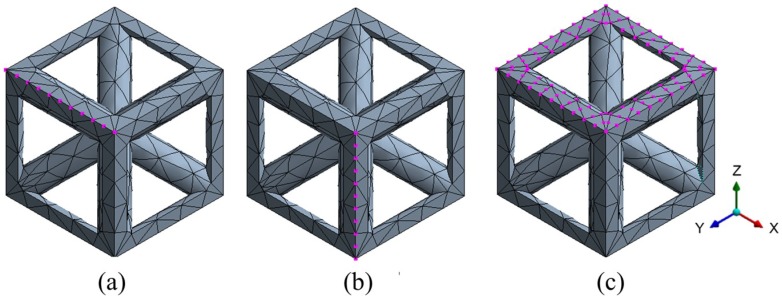
Selections of sets of nodes in a cubic unit cell: (**a**) top left edge nodes, (**b**) front left edge nodes, and (**c**) top face edge nodes.

For each examined lattice type, the FE nodes located at the unit cell edges and faces were identified to allow for the 3D periodicity of the unit cell. Their DOFs were rearranged into a nodal displacement matrix q of the following order

(1) $$q={\left[q_{IN}\;q_{F}\;q_{S}\;q_{B}\;q_{T}\;q_{L}\;q_{R}\;q_{FB\;}q_{FT}\;q_{SB}\;q_{ST\;}q_{FL}\;q_{FR}\;q_{SL}\;q_{SR}\;q_{BL}\;q_{BR\;}q_{TL}\;q_{TR}\right]}^{T},$$

where the subscripts IN, L, R, F, B, T and S denote the DOFs at the inside, left, right, front, bottom, top and back of the single unit cell, respectively (see Figure 6). The displacement matrix $q_{IN}$ contains all the DOFs of nodes that are not located at the unit cell edges and faces. The naming scheme is arbitrary, as it depends on the orientation of the cell; the important principle is the grouping of the node sets according to their location. 

For the application of Bloch’s theorem, a reduced nodal displacement matrix $\bar{q}$ is specified in Bloch’s reduced coordinates [11]. The reduced displacement matrix $\bar{q}$ is used to reduce the stiffness and mass matrices so that they only contain information on the sets of nodes relevant to each examined wavevector. The nodal displacement matrix $\bar{q}$ was obtained by introducing a transformation matrix N of the following shape

(2) $$N=[\begin{array}{ccccccc} I & 0 & 0 & 0 & 0 & 0 & 0\\ 0 & I & 0 & 0 & 0 & 0 & 0\\ 0 & Ie^{-ik_{y}} & 0 & 0 & 0 & 0 & 0\\ 0 & 0 & I & 0 & 0 & 0 & 0\\ 0 & 0 & Ie^{-ik_{z}} & 0 & 0 & 0 & 0\\ 0 & 0 & 0 & I & 0 & 0 & 0\\ 0 & 0 & 0 & Ie^{-ik_{x}} & 0 & 0 & 0\\ 0 & 0 & 0 & 0 & I & 0 & 0\\ 0 & 0 & 0 & 0 & Ie^{-ik_{z}} & 0 & 0\\ 0 & 0 & 0 & 0 & Ie^{-ik_{y}} & 0 & 0\\ 0 & 0 & 0 & 0 & Ie^{-ik_{y}}e^{-ik_{z}} & 0 & 0\\ 0 & 0 & 0 & 0 & 0 & I & 0\\ 0 & 0 & 0 & 0 & 0 & Ie^{-ik_{x}} & 0\\ 0 & 0 & 0 & 0 & 0 & Ie^{-ik_{y}} & 0\\ 0 & 0 & 0 & 0 & 0 & Ie^{-ik_{x}}e^{-ik_{y}} & 0\\ 0 & 0 & 0 & 0 & 0 & 0 & I\\ 0 & 0 & 0 & 0 & 0 & 0 & Ie^{-ik_{x}}\\ 0 & 0 & 0 & 0 & 0 & 0 & Ie^{-ik_{z}}\\ 0 & 0 & 0 & 0 & 0 & 0 & Ie^{-ik_{x}}e^{-ik_{z}} \end{array}]$$

**Figure 7:**
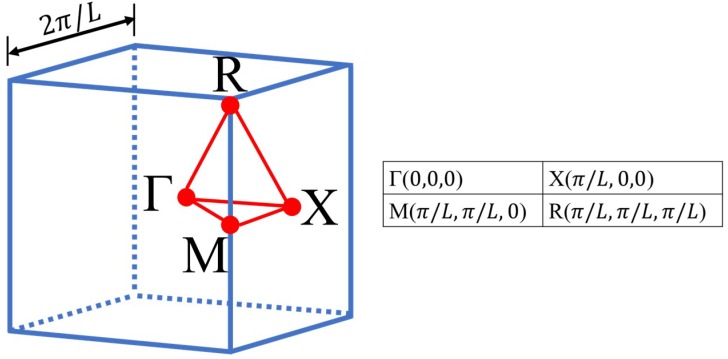
The IBZ of a cubic lattice with the reciprocal space coordinates of the critical points.

Then we have

(3) $$q=N\bar{q},$$

with

(4) $$\bar{q}={\left[q_{IN}\;q_{F}\;q_{B\;}q_{L}\;q_{FB}\;q_{\mathrm{FL}\;}q_{BL}\;\right]}^{T},$$

where k are wavevectors in the irreducible Brillouin zone (IBZ) corresponding to the lattice structure. Projected stiffness and mass matrices of the reduced sets of nodes, $\bar{K}$ and $\bar{M}$ respectively, were then computed using

(5) $$\bar{K}=N^{\prime }KN,$$

and

(6) $$\bar{M}=N^{\prime }MN,$$

where K and M are the global stiffness and mass matrices extracted from the FE model of the single unit cell and rearranged in the same order of q, and $N^{\prime }$ is a transpose matrix used for ensuring force equilibrium [11]. The following eigenvalue problem was then constructed and solved numerically

(7) $$\bar{K}-\omega^{2}\bar{M}=0,$$

where ω denotes the frequency of a propagating wave corresponding to each of the sampled wavevectors. In this work, modelling of wave propagation was restricted to the contour of the IBZ of the examined lattices. According to a statistical study by Maurin et al. [56], restricting the detection of BGs to only the contour of the IBZ rather than the full IBZ provides accurate results for symmetric unit cells as well as savings in computation time. The IBZ of a cubic lattice is shown in Figure 7. 

**Figure 8:**
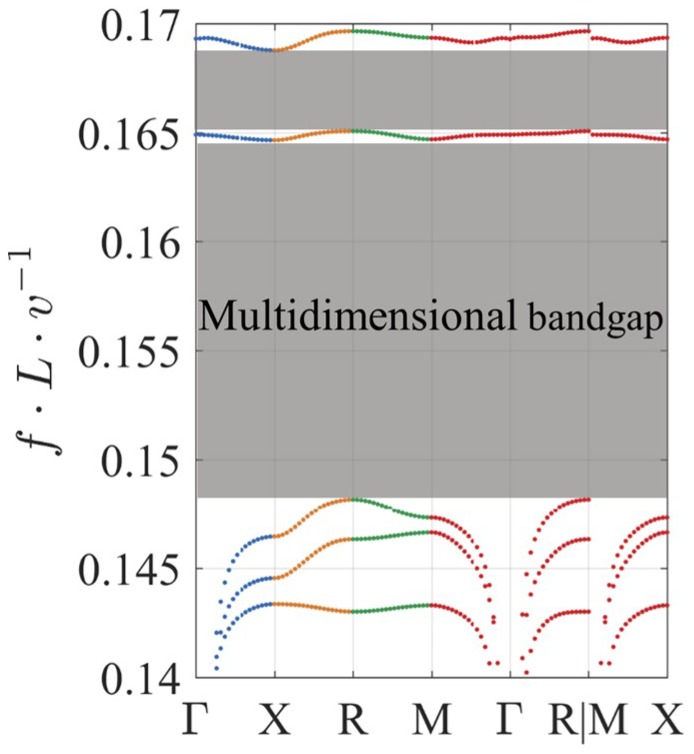
Multidimensional bandgaps identified from calculations of the full contour of the IBZ.

Waves propagating along the path Γ-X, X-R, R-M, and M-Γ of the IBZ were modelled using a total of eighty combinations of wavevectors. The full contour of the IBZ has six paths, however, it is common practice to examine only four of these paths for investigation of multidimensional BGs, as seen in references [13,25,30,31]. Our preparation analysis further reinforced this common practice by showing that waves propagating in paths M-X and Γ-R has minimal/zero effect on the BG position as can be seen in Figure 8. The frequency eigenvalues of Equation (7) were normalised to the unit cell size L and the speed of the wave in the lattice material v to obtain normalised frequencies. The dispersion curves (DCs) were then extracted as corresponding pairs of wavevector and normalised frequency.

The DC calculations make use of infinite periodic boundary conditions in the FE models. In practical applications, only lattice structures of finite periodicity are realisable (i.e., manufacturable). This limitation is expected to modify the DCs and reduce the extent of vibration attenuation achievable with these lattices. Thus, in Section 3.4, we calculated the transmission of waves through lattice structures with very low periodicity. The choice of unit cell for this study was made from the unit cells that exhibited BGs under infinite periodic boundary conditions, as depicted by their respective DCs. The transmission of waves in the selected lattices was conducted using ANSYS Workbench. Longitudinal (primary) waves were modelled passing through the lattice structures. The wave transmission between the input side and the opposite side of the lattice was determined with a normalised frequency resolution of 0.0025.

For all calculations, the lattice structures were modelled with the properties of L-PBF Nylon-12, which are shown in Table 4. The choice of this material is based on its compatibility with the L-PBF fabrication process. Nylon-12 has a large temperature processing window that allows for uniform crystallisation during cooling of the part; thus leading to reduction of material warpage and lamination [57], which is essential for manufacturing parts with predictable geometries and mechanical properties. Details about the physics and the challenges of L-PBF can be found elsewhere [58].

## 3. Results and Discussion

### 3.1. Verification of the Dispersion Curve Calculations

**Figure 9:**
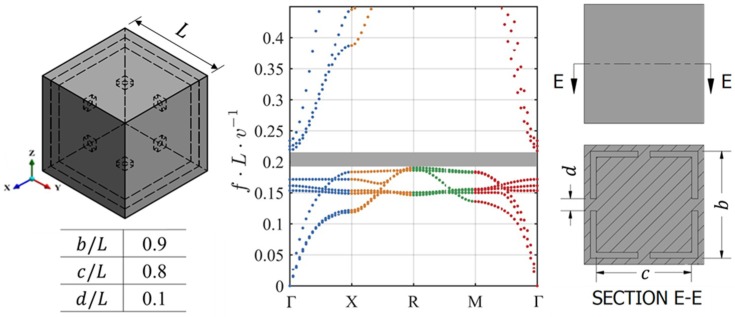
Multidimensional dispersion curves (DCs) of the lattice proposed by Wang et al. [25] as remodelled using our finite element (FE) modelling technique. The shaded grey area in the DC plot represents the identified bandgap (BG).

The FE wave modelling technique, detailed in Section 2.2, was used to calculate the DCs of the lattice examined by Wang et al. [25] for verification purposes. This lattice was selected due to its CAD modelling simplicity and clear BG. The lattice is modelled in CAD using the design parameters in Figure 9 and a Poisson’s ratio of 0.33.

As can be seen in Table 5, the DCs resulting from the FE method employed in this work exhibited a very similar BG to that predicted by Wang et al. The difference of 0.01 in the normalised BG end frequency is likely due to the difference in our respective meshing techniques (Wang el al. did not report an exact mesh density) and BG identification methods. 

### 3.2. Wave Dispersion in Lattices with Infinite Periodicity

**Figure 10:**
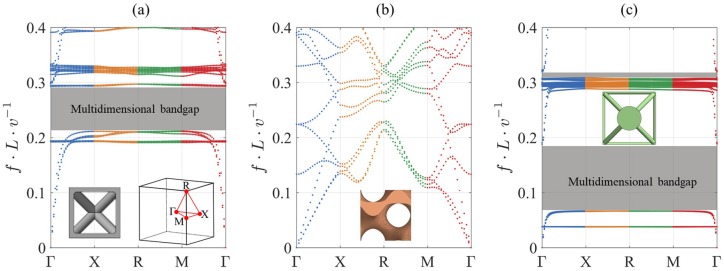
DCs of (**a**) BCC_xyz_, (**b**) gyroid TPMS, and (**c**) res-BCC_xyz_ lattice structures with 20% volume fraction.

The wave propagation dispersion curves (DCs) of the three considered lattice types, all with 20% volume fraction, are shown in Figure 10. Multidimensional BGs are identified in the DCs of the BCC_xyz_ and res-BCC_xyz_ lattices. The gyroid TPMS lattice did not show a multidimensional BG, although it is known to exhibit a 1D BG [18]. Below a normalised frequency of 0.2, the res-BCC_xyz_ lattice was the only lattice that showed a BG. Above a normalisd frequency of 0.2, both the BCC_xyz_ and res-BCC_xyz_ lattices possess one BG. The first BG of the res-BCC_xyz_ lattice is wider by 57% and has a BG starting frequency lower by 68.5% than that of the BCC_xyz_ lattice. The BG frequency width (bandwidth) of the BCC_xyz_ lattice is eight times that of the second BG of the res-BCC_xyz_ lattice.

In general, lattices that are manufacturable with current AM methods, and have broad BGs with low starting frequencies are the most desirable. This is because they can be tailored for use in various applications, including precision engineering and metrology, providing a wide frequency range over which vibration transmission is restricted. In comparison, if the intrinsic BG frequency of a particular lattice type is high, efforts to tune its frequency by modifying the cell size will generally result in unrealistic or unmanufacturable cell sizes [15]. 

A relative gap to mid-gap ratio (relative BG width) can be used to compare the calculated BGs of the BCC_xyz_ and res-BCC_xyz_ lattices to those of the BCC-inspired multimaterial lattices of Lu et al. [13] and Husieh et al. [45]. A relative BG width is the quotient of the bandwidth and the BG mean frequency (BGMF) and is independent of the unit cell size; a high relative BG width is more desirable as it indicates a wide BG with low frequency. The BG of Lu et al. had a relative width of ~60%, while that of Husieh et al. was ~40%, both at a volume fraction of ~23%. Interpolation of our BG results for the BCC_xyz_ and res-BCC_xyz_ lattices at 23% volume fraction showed a relative BG width of 30% and 98.7%, respectively. This indicates that the res-BCC_xyz_ lattice has the ability to provide wide BGs of low starting frequencies using single material lattices.

The multidimensional BG of the BCC_xyz_ lattice is compared to the 1D BG of the gyroid TPMS, which was studied by Elmadih et al. [18]. At similar volume fraction, the BCC_xyz_ lattice shows BGs at higher frequencies than the gyroid TPMS lattice. For example, at 20% volume fraction, several BGs are present below a normalised frequency of 0.2 in the gyroid TPMS lattice. However, the bandwidth of this BCC_xyz_ lattice is almost five times wider than that of the 1D gyroid TPMS BG.

**Figure 11:**
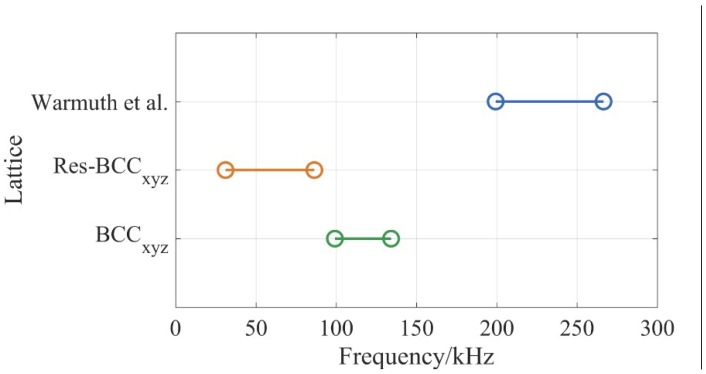
BG properties of the BCC_xyz_, res-BCC_xyz_ and Warmuth et al. [30] lattices of 20% volume fraction and 10 mm unit cell size, as predicted using the material properties of Ti-6Al-4V.

The normalised results in Figure 10 can be used to predict BGs in BCC_xyz_ lattices of various materials and unit cell sizes. For example, BGs of Ti-6Al-4V BCC_xyz_ lattices can be predicted. Ti-6Al-4V is used in the aerospace and the biomedical sectors due to its high corrosion resistance, biocompatibility and high fracture toughness [60]. The phononic properties of Ti-6Al-4V strut-based lattices have been studied previously [30]. For the purpose of comparison with the BCC_xyz_ and res-BCC_xyz_ lattices presented here, a unit cell 10 mm in size and 20% volume fraction, based on the design of Warmuth et al. was considered. Using the BG tuning tool in Equation (1) from [30], the BG properties were calculated. The BG starting frequency and BG ending frequency of the BCC_xyz_, res-BCC_xyz_ and the Warmuth et al. lattice of 10 mm unit cell size and 20% volume fraction are presented in Figure 11. The relative BG width of the BCC_xyz_ and res-BCC_xyz_ lattices at 20% volume fraction were 30% and 94%, respectively, while the relative BG width of the Warmuth et al. structure is 28.8% at the same volume fraction. The BG of the BCC_xyz_ lattice has a lower bandwidth than the lattice of Warmuth et al.; the BG of the BCC_xyz_ lattice spanned 99.2 kHz to 134.2 kHz, which is approximately 52% of the bandwidth of the lattice of Warmuth et al. However, the BCC_xyz_ lattice had the ability to provide BGs at frequencies lower by 50.2% than those of Warmuth et al. at similar cell size and volume fraction.

### 3.3. Tuning of Multidimensional BGs

For a range of volume fractions, the properties of the lowest frequency BG were identified from the DCs of the BCC_xyz_ and res-BCC_xyz_ lattices. The 5% and 10% volume fraction BCC_xyz_ lattices showed two BGs below a normalised frequency of 0.4. The lowest frequency BG of the 5% volume fraction BCC_xyz_ lattice spanned a normalised bandwidth of 0.014, from 0.151 to 0.165. This BG is the narrowest in width and is formed by an acoustic waveband (wave cutting-on at zero frequency) and an optical waveband (wave cutting-on at a non-zero frequency). However, 20% and 30% volume fraction BCC_xyz_ lattices had no second BGs. 

**Figure 12:**
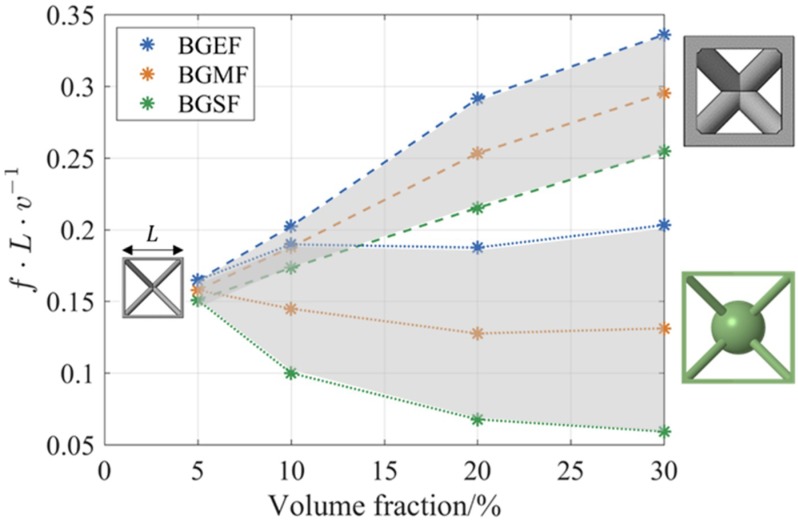
Attributes of the BGs as identified from the DCs of the BCC_xyz_ (dashed lines) and res-BCC_xyz_ (dotted lines) lattices at different volume fractions.

The BCC_xyz_ lattice of 30% volume fraction showed the highest predicted BG, which spanned from 0.25 to 0.34. The res-BCC_xyz_ lattice of 30% volume fraction showed the lowest predicted BG, which spanned from 0.067 to 0.187. The res-BCC_xyz_ lattice has a BGMF lower by an average of 43% than the BGMF of the BCC_xyz_ lattice, as calculated from the BGMF of lattices with volume fractions of 5% to 30%. The bandwidth increased approximately fivefold, and tenfold upon increasing the volume fraction of the BCC_xyz_ and res-BCC_xyz_ lattices, respectively, from 5% to 30% as can be seen in Figure 12. 

Bragg-scattering BGs are bounded by a natural frequency of the Bragg scattering unit cell (BCC_xyz_) [14], while internal resonance BGs (res-BCC_xyz_) occur around the natural frequency of the internal resonance mechanism [61]. Thus, the two BG mechanisms can be explained by referring to the natural frequency $f_{n}$ equation, $f_{n}\propto \sqrt{k/m}$. Above a volume fraction of 5%, additional material uniformly enlarges the struts of the BCC_xyz_ lattices, which results in stiffer lattices of higher mass. It has been shown that the BG frequency increases with the increase in volume fraction of Bragg-scattering BGs lattices [13,18]; indicating that the stiffness increases at a greater rate than the rate at which the mass increases. The BG mechanism for the res-BCC_xyz_ lattice is different since the mass of the resonance mechanism is dictated by the mass of the solid sphere, while the stiffness is dictated by the stiffness of the resonance struts. Since above a volume fraction of 5% additional material enlarges the size of the sphere of the res-BCC_xyz_ lattices, but does not increase the diameter of the struts, an overall reduction of the natural frequency of the resonance mechanism is achieved, thus, reducing the internal resonance BG frequency. 

### 3.4. Evolution of the Wave Transmission in Lattices with Finite Periodicity

**Figure 13:**
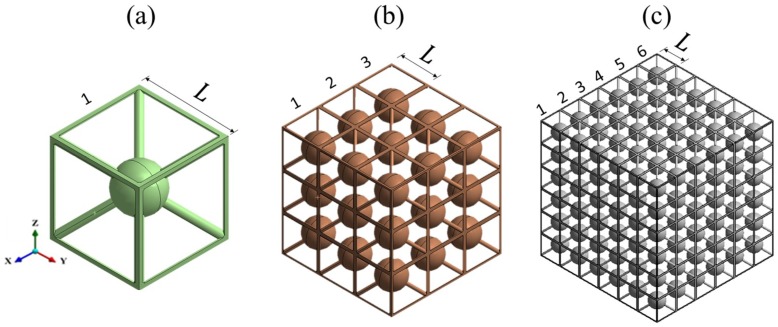
Res-BCC_xyz_ lattice structures of finite periodicities of (**a**) one, (**b**) three and (**c**) six.

As detailed in Section 2.2, the transmission spectrum of lattice structures with different periodicities were modelled to examine the evolution of the BG in physically realisable components. The res-BCC_xyz_ lattice at 20% volume fraction was selected for this study. This lattice was found to have a wide BG spanning normalised frequencies from 0.067 to 0.187, as seen in Figure 12. The examined lattice structures, shown in Figure 13, had periodicities of one, three and six (i.e., they contained a single unit cell, 3 × 3 × 3 and 6 × 6 × 6 cells, respectively).

**Figure 14:**
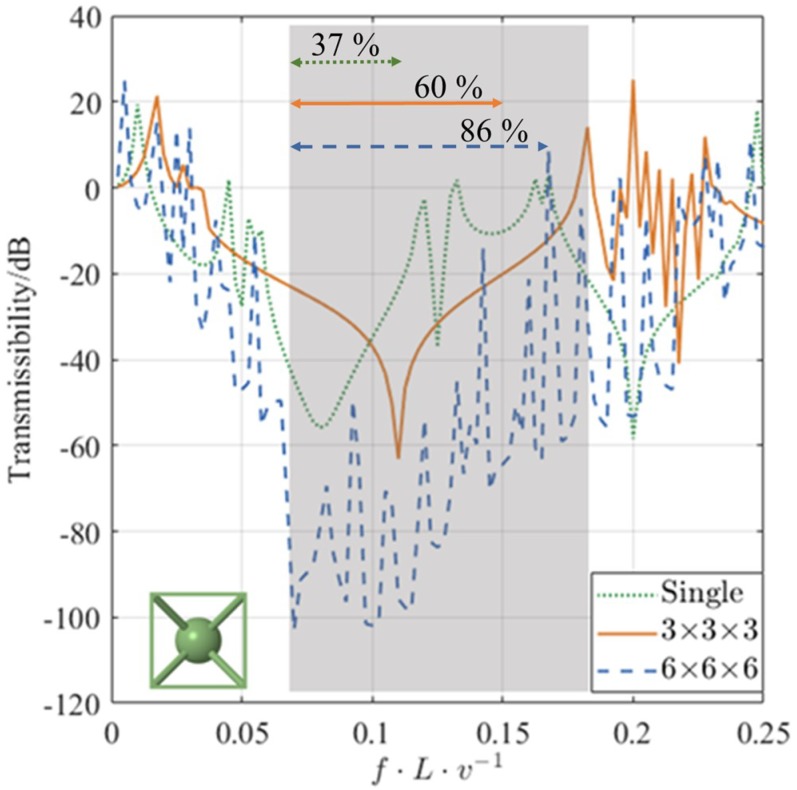
Transmissibility of longitudinal waves in 20% volume fraction res-BCC_xyz_ lattices of finite periodicity. The shaded area represents the BG region as depicted by the DCs with infinite periodicity assumptions. The percentage values denote the bandwidth of the finite lattice to that of the infinite one.

Outside the infinite BG, the transmission of the waves in the finite lattices varied between ±20 dB as can be seen in Figure 14. For identification of BGs, it seems reasonable to use −20 dB as the upper limit for the transmission within a BG. For the single unit cell, the BG was shown between normalised frequencies of 0.06 to 0.11; this bandwidth is narrow (only 37% of that identified from the DCs). The narrow bandwidth can be traced back to the lack of spatial periodicity, which is essential for obtaining transmission BGs. The width of the BG for the 3 × 3 × 3 lattice is higher than that of the single unit cell and spanned from 0.06 to 0.15; this bandwidth is 60% of that identified from the DCs of the infinite lattice. The largest BG, with the assumption of −20 dB as the upper amplitude limit, is for the 6 × 6 × 6 lattice which spanned 86% of the bandwidth identified from the DCs of the infinite lattice. In addition to the change in width, the minimum and mean amplitudes within the infinite BGs also changed with the periodicity of the finite lattice. Table 6 summarises the mean and minimum transmissibility of longitudinal waves in finite lattices of various periodicities. As the lattice periodicity increases, the transmissibility within the BG decreases.

## 4. Conclusions

Reported here is an investigation into the potential for 3D AM lattice structures to provide multidimensional BGs for the purpose of vibration isolation. The BGs were identified from the structures’ dispersion curves calculated using a FE-based wave propagation modelling technique and infinite periodic boundary conditions. The FE technique provides high computational efficiency and high wave modelling accuracy. Key results included:

- Single material BCC_xyz_ and res-BCC_xyz_ lattices can provide BGs that are tunable with the volume fraction of the lattice.
- BCC_xyz_ and res-BCC_xyz_ lattices have BGs of high width and intrinsically low frequency compared to results reported for similar structures in the literature.
- Although gyroid TPMS lattices are known to have 1D BGs, they do not exhibit multidimensional BGs.
- An increase in the finite periodicity of the lattice leads to an increase in the bandwidth and to a decrease in transmissibility within the BG.
- The attenuation of longitudinal waves reaches a minimum of −103 dB within the BG.

These results complement the set of design tools already available for AM parts, which are mainly concerned with the static support and load-bearing properties, by adding a tuning tool for enhanced mechanical vibration isolation. 

## Figures and Tables

**Table 1:** Design data of multiple BCC_xyz_ lattices.

Volume Fraction (%)	d/L
5	0.084
10	0.121
20	0.178
30	0.226

**Table 2:** Design data of multiple gyroid TPMS lattices.

Volume Fraction (%)	d/L
5	0.138
10	0.175
20	0.250
30	0.325

**Table 3:** Design data of multiple res-BCC_xyz_ lattices.

Volume Fraction (%)	d/L	s/L
10	0.084	0.480
20	0.084	0.680
30	0.084	0.796

**Table 4:** Properties of L-PBF Nylon-12 used for modelling lattice structures in this work [59].

Tensile Modulus	Density	Poisson’s Ratio
1500 MPa	950 kg·m^−3^	0.3

**Table 5:** BG properties as identified in this work and reported by Wang et al. [25].

BG Property	Remodelled Structure (This Work)	Wang et al. ([25])
Normalised BG start frequency	0.19	0.19
Normalised BG end frequency	0.22	0.23
Normalised BG frequency width (bandwidth)	0.03	0.04

**Table 6:** Summary of the evolution of the BG as obtained from studying the transmissibility of longitudinal waves in BG lattices of different periodicity.

Periodicity	Mean Transmissibility (dB)	Lowest Transmissibility (dB)
One	−23	−56
Three	−24	−63
Six	−66	−103
